# The landscape of undergraduate public health writing instruction: a qualitative assessment

**DOI:** 10.3389/fpubh.2026.1737041

**Published:** 2026-04-01

**Authors:** Uday Patil, O. Vanessa Buchthal, Elizabeth McFarlane, Eric L. Hurwitz, Betsy Gilliland, Katherine I. Yang, Denise C. Nelson-Hurwitz

**Affiliations:** 1 Department of Public Health Sciences, Thompson School of Social Work and Public Health, University of Hawai‘i at Mānoa, Honolulu, HI, United States; 2 Department of Second Language Studies, University of Hawai‘i at Mānoa, Honolulu, HI, United States

**Keywords:** bachelor’s in public health, public health, public health pedagogy, undergraduate education, writing instruction, writing skills

## Abstract

Strong writing skills are arguably the most useful and marketable trait of a graduating public health student. This study reviewed the national landscape for approaches to integrating writing skill development into undergraduate public health curricula with implications for the development of effective writing pedagogy within public health programs. Leaders of all 100 Council on Education for Public Health-accredited undergraduate public health schools, programs, and independent units in the US were invited to participate in interviews and provide information on student writing challenges. A total of 30 staff or faculty participated in this semi-structured interview between November 2022 and February 2023. Half also provided documents for additional review to complement the interviews. Information shared by interviewed participants was coded and organized into six categories by researchers: approaches, assignments, support, concepts, context, and needs. Based on interview data and complementary documents, instructional commonalities identified among programs included a wide variety of writing assignments administered in courses, such as literature reviews and needs assessments. In contrast, practices supporting student success are less varied, and frequently include repeated reading, parsing, and writing scientific articles. These activities have meaningful effects on students and are complementary to prolonged practice in managing and using citations. Many pedagogical approaches can be taken when guiding students to build those skills, including the high-impact educational practice of scaffolding. Findings were placed within a framework of considerations for writing skill instruction in undergraduate public health curricula. The developed framework has implications for improved understanding and broad-based context related to pedagogical practice of writing instruction in undergraduate public health, with implications for improved student support, faculty instruction, and bachelors program curricula.

## 1 Introduction

Writing is integral to public health and the greater health sciences (1, 2). Public health professionals are expected to communicate important findings authoritatively but also with rhetorical passion and strength of argument to win over peers and laypersons alike (3, 4).

While broad, overarching models and frameworks for public health writing pedagogy are lacking, established researchers have developed varied approaches to, and tools for, teaching public health writing. Among existing literature, Beard and colleagues have described best practices in public health writing pedagogy (5, 6) in addition to developing a peer writing coach program (7), collegiate public health writing program (8), and various tools for instructors and students alike (6, 8) as applied in a large university. Similarly, Mackenzie (9) also expertly documented approaches to teaching writing and integrating the previously established practice of writing across the curriculum (WAC) (10–12) within a single public health school. These contributions are meaningful to individual instructors of public health writing, but lack macroscale evidence of existing public health writing practices and assignments across multiple public health programs, which may serve to guide the integration of writing skill development in public health studies.

The development of a broad academic consensus and pedagogical models specific to public health writing is further complicated as individual public health instructors often disagree on techniques, practices, and criteria to be employed when teaching writing (9, 13–15). This variability contributes to a widening gap in consistency of public health writing instruction, as few instructors report access to support for writing instruction (2, 15). One consistency among most faculty is acknowledgement of a problem with the current state of public health student writing (2). Similarly, the Council on Education for Public Health (CEPH) added criteria requirements for written communication to its 2016 revised Accreditation Criteria for Schools of Public Health and Public Health Programs (current criteria D9- domain 11, and D10) (16). This emphasizes a clear need, but leaves the standard open to be met using variable approaches developed by individual degree programs (17). A lack of standards for what courses, exercises, and assignments should look like, and related persistence of poor writing skills, leads to students being inadequately prepared for post-graduate work and ill-prepared to meet workforce needs.

Without clear, agreed-upon writing standards, fostering writing skill development in the next generation of public health professionals is difficult. Meanwhile, in the context of growing public health workforce shortages (18–22), deficiencies in public health professional writing skills may further constrain workforce effectiveness. To summarize the issue, if public health undergraduates cannot write effectively, they cannot fulfill critical roles during a time when the workforce is already diminished.

To improve public health undergraduate writing, and subsequently strengthen ability to meet workforce needs, further examination of public health writing instruction is a necessary step toward reducing identified skill gaps in written communication, as identified by previous studies (23–25) and workforce training needs assessment data (26). Discipline-specific writing skills are often taught through a variety of written assignments embedded in one or more undergraduate courses within a degree program of study. These assignments and associated courses are strong areas for further examination. Identifying how and where written assignments are used, and the degree of effectiveness associated, may lead to an improved understanding of how writing skills, especially foundational process-oriented skills, are developed in undergraduate public health programs and where gaps may exist. A comprehensive survey of the landscape of writing instruction among public health undergraduate degrees is necessary for understanding the variety of assignments, embedded writing curricula, and effectiveness of writing instruction practices, beyond standardized reporting for accreditation. The implications of such examination have the potential to benefit individual programs in meeting student needs and accreditation standards, while working towards improving the writing skills of the future public health workforce.

### 1.1 Study purpose

This study qualitatively reviewed undergraduate public health programs via interview and document review, intending to create a framework of considerations to support writing instruction broadly and to guide instructors in the improvement of student writing.

## 2 Methods

This study collected data directly from all CEPH-accredited schools, programs, and independent units granting undergraduate public health degrees in the US (100 in total as of February 2022). Institutions were identified by a list of accredited schools, programs, and independent units available on the CEPH website (27). Degree program points-of-contact were identified, and contact information for recruitment was collected, from the websites of degree programs as linked on the CEPH website. Points-of-contact were recruited through a maximum of three email outreach efforts during the study period. Points-of-contact as identified on program websites included program directors, department chairs, and other leaders affiliated with undergraduate programs of public health. All were solicited for participation themselves, or were invited to recommend someone in their program to interview about students’ writing skills development in their program. If a referral was made, recruitment efforts were made by email to that individual. Participants were interviewed virtually and solicited for supporting documents.

Questions for the semi-structured interviews were developed collaboratively by the first and last authors following review of existing literature, and several key articles in particular identified by authors during the literature review (6, 9, 15, 28), and based on the authors’ writing instruction experience developed through instruction of multiple undergraduate public health courses at the University of Hawai‘i at Mānoa. While questions were both theory-driven and practice-driven, theory guided initial development with practice informing additional details and areas identified for further question probing. Interview questions were then reviewed with minor clarifying revisions made by the second and fifth authors, both experienced qualitative researchers.

As related to reflexivity, authors are interested in promoting post-secondary education. Authors are familiar with post-secondary writing processes and associated literature, and also have over 55 years of combined experience in undergraduate instruction, particularly as related to writing skill development.

The interview and data collection protocols were pilot-tested with the undergraduate staff and faculty of the Department of Public Health Sciences at the University of Hawai‘i at Mānoa, and data collection began in November 2022 after minor revision.

### 2.1 Human subjects research

This study was reviewed and approved by the University of Hawai‘i Human Studies Program, as exempt from federal regulations pertaining to the protection of human research participants (Protocol #2022-00624).

### 2.2 Eligibility criteria

This study solicited participation from all 100 members of a complete list of CEPH-accredited schools, programs, and independent units granting undergraduate public health degrees, as seen in Appendix A (17).

### 2.3 Data solicitation

Data collection included both virtual interviews and a complementary document analysis of content solicited from program directors, department chairs, and other leaders affiliated with undergraduate programs of public health at identified programs, and any contextual notes or opinions collected from the data provider. Requested documents included assignment instructions, course syllabi, program curricula, and evaluation notes.

The first author e-mailed degree programs’ points-of-contact as identified by CEPH to gather data or referrals to individuals more informed of the degree program’s efforts to promote writing instruction. Three email contact attempts at recruitment were made by email, once per month, between November 1, 2022 and January 31, 2023. The recruitment time period was identified to span a brief portion of both fall and spring semesters, for schools utilizing the semester system, and to encompass holiday or winter break time periods, for schools that observe, where participants may be more available for study participation. The study period also reflected limited time availability of researchers for data collection.

The objective of email correspondence was to submit data requests and to schedule a Zoom call to retrieve informed consent, follow up on data requests, and collect contextual information regarding the degree-granting program.

### 2.4 Data collection and synthesis

Interviews with participants were conducted via Zoom by the first author between November 2022 and February 2023. Completed interviews ranged from 30 to 75 min, including IRB consent procedures and solicitation for any complementary documents. Only typed or handwritten notes were collected by the interviewer. Notes were documented in real time during the interviews, and summarized immediately following to ensure accuracy and minimize bias. No recordings or transcripts were made, which is a meaningful limitation. Regular check-ins were conducted with the last author to discuss progress and ensure method consistency.

Requested documents included document types suited for public health/epidemiology assignments, such as journal articles of original research, literature reviews, system evaluations, grant proposals, annotated bibliographies, and issue briefs (13). In a 2012 survey of documents commonly used in public health by the University of Washington School of Public Health, 19 forms were identified, adding abstracts, health alerts, health promotion brochures, status reports, nonfiction monographs, op-eds, position papers, posters, press releases, protocols, public service announcement, slides, textbooks, and white papers (9). Materials were identified and shared, given this context, by email at the discretion of the interview participant either during or following the interviews. Providers of collected data were asked to provide relevant contextual information and opinions on efficacy and usefulness of instructional practices, particularly those described in provided materials, during interviews. Usefulness of materials provided was enhanced by context discussed in the interviews. While materials directly provided documentation of educational practices and logistical information, context provided during interviews often discussed frequency and potential scaffolding of application, student responses to tools and instruction, and perceived usefulness. For example, a provided course schedule may illustrate components of how a large paper may be split, or broken down, into smaller assignments, while interview data addressed high effectiveness of this practice, and individual component assignments where students were more challenged than others.

**Figure 1:**
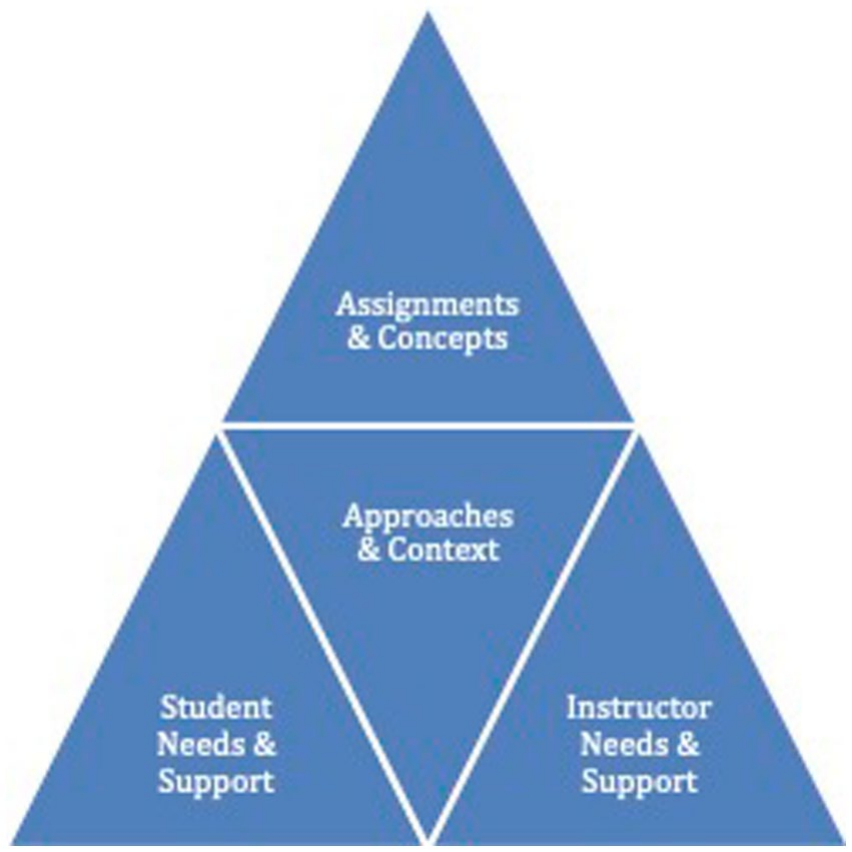
Instructional design considerations.

Qualitative content analysis was employed to synthesize information and perspectives presented in the interviews and Supplementary materials (29–31). All materials shared by participants were mined according to the O’Leary guide to document analysis (32). Document contents, related contextual information, and participants’ opinions on efficacy and usefulness as expressed in the interviews were coded 479 times to summarize and classify content. 139 codes were used to classify information and were organized into categories, themes, and groups representing meaningful patterns. Top-level categories represented general areas related to instructional practices and needs (represented in Figure 1). Themes referred to more specific categories of information within each category. In some themes, code groups were used to cluster lowest-level codes describing related practices. The lowest-level codes signified specific examples of practices endorsed by participants or exemplified in the materials. In this context, endorsement refers to participants verbally stating that they engage in the activity or idea as prompted during the interview, or the activity was evident in the documentation provided by participants, as identified during document review.

The first author developed the coding scheme in a hybrid approach: deductive codes were initially identified in the reviewed literature, and additional inductive codes were developed when concepts emerged from the data during analysis. Upon completion of the analysis, the results, proposed codes, and themes were reviewed by three co-authors of the study to minimize bias and establish concordance among co-authors. Any disagreements on codes and themes were resolved by siding with the majority or at the discretion of the first author in the event of a split decision (29–31).

### 2.5 Participants

As of February 2022, CEPH reported 100 accredited universities, colleges, or standalone programs in the United States offering undergraduate public health degrees. This did not include unaccredited public health programs or programs pending accreditation. Programs offered Bachelor of Science (BS), Bachelor of Science in Public Health (BSPH), Bachelor of Public Health (BPH), Bachelor of Arts (BA), or Bachelor of Arts in Public Health (BAPH) degrees (17).

The researcher contacted a staff or faculty member point of contact, as reported by CEPH, at each of 100 undergraduate public health programs as described above. During the recruitment period of November 2022 to January 2023, representatives from 30 programs responded and agreed to participate. The decision to cease recruitment was made following a collaborative discussion of co-authors most familiar with qualitative data collection. The decision was informed by the diversity of responding programs (e.g., geographical and program size variability), the time available for data collection, and the conclusion of three email contact attempts for participation. The decision to cease recruitment was revisited following data analysis, and was confirmed based on clustering of findings and time constraints of researchers. All 30 representatives who responded were interviewed between November 2022 and February 2023.

Fifteen (50%) provided complementary documents for review. A total of 47 documents were collected from 15 participants. Syllabi (*n* = 20, 43%) or assignment instructions, guidelines, and rubrics (*n* = 17, 36%) accounted for the majority of reviewed documents. The remaining documents were sample student work, rubrics, source material guiding curriculum development, course crosswalks, and program overviews.

Most participating programs (*n* = 22, 73%) were located on the east coast of the US, with 10 (33%) in the Northeast, and 12 (40%) in the Southeast with three programs each from Florida, Georgia, and North Carolina. Of the remaining eight programs, five (17%) were located in the Midwest—two (7%) in Illinois and Kentucky each. Three (10%) programs were located on the west coast. Of those, two (7%) were located in California. In comparison, among all 100 CEPH-accredited universities, colleges, or standalone programs in the United States offering undergraduate public health degrees, programs are most frequently located in the Southeast (*n* = 39), followed by the Northeast (*n* = 22), West (*n* = 20), and Midwest (*n* = 19).

Almost half (*n* = 14, 47%) of the participating programs only offered a BS degree option, while nine (30%) other programs only offered a BSPH degree. Four (13%) programs offered students a choice between BA and BS degree pathways. Three other schools offered a combination of the degree choices listed above or similar degrees, like a Bachelor of Science in Health or a Bachelor of Health. In comparison, across U.S. CEPH-accredited undergraduate programs, 168 degree offerings were identified, including BS (*n* = 75), BSPH (*n* = 61), BA or BA/BS pathways (*n* = 24), and a smaller number of related or combined degree designations (*n* = 8).

Geographic and programmatic variation of participants compared to that of all U.S. CEPH-accredited undergraduate programs are summarized in Table 1.

**Table 1:** Geographic and programmatic variation of study sample vs. all CEPH-accredited programs.

	Study participating programs	All U.S. CEPH-accredited programs
Number of programs	30	100
U.S. geographic area		
Southeast	12	39
Northeast	10	22
Midwest	5	19
West	3	20
Degrees offered		
Bachelor of Science (BS)	14	43
Bachelor of Science in Public Health (BSPH)	9	34
Bachelor of Arts (BA) or BA/BS Pathways	4	6
Combination of Degrees/Related Degrees	3	17

## 3 Results

### 3.1 Codes and themes

Following interviews and document review, six top-level coding categories were identified: Approaches, Assignments, Support, Concepts, Context, and Needs. See Table 2 for the codes and respective endorsement rates by participants.

**Table 2:** Frequencies and percentages of theme and code endorsement (*n* > 4).

Category	Theme	Code group	Code	*n*	%
Approaches: Which pedagogical approaches did instructors (*n* = 30) take?				29	96.7%
Approaches	Classroom			29	96.7%
Approaches	Classroom		Group work	9	30.0%
Approaches	Classroom		Low-stakes approach	6	20.0%
Approaches	Classroom		Multiple drafts	10	33.3%
Approaches	Classroom		Peer review	6	20.0%
Approaches	Classroom		Scaffolding	18	60.0%
Approaches	Classroom		Staged writing	13	43.3%
Approaches	Curricular			22	73.3%
Approaches	Curricular		Confidence building	6	20.0%
Approaches	Curricular		Feedback provision	7	23.3%
Approaches	Curricular		Language & style flexibility	5	16.7%
Approaches	Curricular		Writing across the curriculum approach	5	16.7%
Assignments: Which assignments did instructors give?				21	70.0%
Assignments	Discipline-specific Instruction			20	66.7%
Assignments	Discipline-specific Instruction	Literature reviews		15	50.0%
Assignments	Discipline-specific Instruction	Literature reviews	Literature review (brief)	7	23.3%
Assignments	Discipline-specific Instruction	Literature reviews	Literature review (full)	11	36.7%
Assignments	Discipline-specific Instruction		Needs assessments	7	23.3%
Assignments	Discipline-specific Instruction		Policy analysis briefs	5	16.7%
Assignments	Discipline-specific Instruction		Research proposals	5	16.7%
Assignments	Writing instruction			17	56.7%
Assignments	Writing instruction		Article summaries	8	26.7%
Assignments	Writing instruction		Professional portfolio	6	20.0%
Assignments	Writing instruction		Reflections	5	16.7%
Concepts: Which concepts and topics did instructors teach?				26	86.7%
Concepts			Abstracts and summaries	9	30.0%
Concepts			Academic literature reading	9	30.0%
Concepts			Analysis and synthesis	13	43.3%
Concepts			Audience, purpose, and tone	9	30.0%
Concepts			Citations and references	10	33.3%
Concepts			Forming arguments	5	16.7%
Concepts			Rhetoric and composition	5	16.7%
Concepts			Technical content integration	11	36.7%
Context: In which courses did instructors require long written assignments?				23	76.7%
Context			Capstone	6	20.0%
Context			Introduction	13	43.3%
Context			Research methods	8	26.7%
Context			Social and behavioral health	5	16.7%
Context			Social determinants of health	5	16.7%
Needs: Which needs do instructors have?				19	63.3%
Needs	Redesign			7	23.3%
Needs	Redesign		General education requirements	6	20.0%
Needs	Training			5	16.7%
Support: Which supporting services and tools do instructors have?				26	86.7%
Support	Services			26	86.7%
Support	Services		Library	5	16.7%
Support	Services	Writing center		22	73.3%
Support	Services		Writing workshops	5	16.7%

Within the Approaches category, 35 unique, lowest-level codes were endorsed by 29 of 30 (97%) participants. Participants endorsed classroom-related strategies such as scaffolding (*n* = 18, 60%) and splitting assignments (*n* = 13, 43%), and curricula-related strategies including feedback provision (*n* = 7, 23.3%) and confidence building (*n* = 6, 20%).

Within the Assignments category, 24 unique, lowest-level codes were endorsed by 21 (70%) participants. Participants mentioned not only discipline-specific instruction information such as literature review teaching strategies (*n* = 15, 50%) and needs assessment tips (*n* = 7, 23%) but also writing-specific instruction information (*n* = 17, 57%) like article summary, technical document, and reflection teaching strategies.

Within the Support category, 20 unique, lowest-level codes were endorsed by 26 (87%) participants. Participants mentioned the value of campus-wide writing centers (*n* = 22, 73%), other library support (*n* = 5, 17%), writing workshops (*n* = 5, 17%), teaching assistant policies (*n* = 4, 13%), and wraparound services and policies (*n* = 4, 13%).

Within the Concepts category, 20 unique, lowest-level codes were endorsed by 26 (86.7%) participants. Participants (*n* = 13, 43%) spoke or wrote about the difficulty their students had analyzing and synthesizing data; integrating technical content (*n* = 11, 37%); citing sources (*n* = 10, 33%); understanding audience, purpose, or tone (*n* = 9, 30%); reading academic literature (*n* = 9, 30%); and summarizing information (*n* = 9, 30%).

Within the Context category, 19 unique, lowest-level codes were endorsed by 23 (77%) participants. Participants mentioned courses where writing instruction was happening. The most common were introductory courses (*n* = 14, 47%), courses on research methods (*n* = 8, 27%), or part of a capstone sequence (*n* = 6, 20%).

Within the Needs category, 18 unique, lowest-level codes were endorsed by 19 (63%) participants. As reported by the participants, staff and faculty requested a curriculum overhaul or redesign (*n* = 7, 23%), mostly of general education requirements and not public health degree programs; additional training (*n* = 5, 17%), mostly for teaching writing in the health sciences; additional instructional resources (*n* = 3, 10%); and enhanced program monitoring and evaluation (*n* = 3, 10%).

### 3.2 Research products

Codes and themes from qualitative data analysis informed a framework of considerations when designing an effective writing-intensive course within an undergraduate public health program. See Table 3 for the framework of considerations, which lists the most prominent findings by category, in order of popularity.

**Table 3:** Framework of considerations when designing writing-intensive courses in order of popularity.

Category	Frequently endorsed themes
Approaches	Scaffolding
	Splitting assignments
	Providing feedback
	Building confidence
Assignments	Literature reviews
	Writing-specific exercises
Concepts	Difficulty analyzing and synthesizing data
	Integrating technical content
	Citing sources
	Understanding audience, purpose, or tone
	Reading academic literature
	Summarizing information
Context	Introductory courses
	Research methods courses
	Capstone courses
Needs	Curriculum redesign
	Additional instructor training
	Additional instructional resources
	Enhanced program monitoring
Support	Writing centers
	Library support
	Writing workshops
	Teaching assistants
	Wraparound services

In the framework, each of the six categories identified (Approaches, Assignments, Support, Concepts, Context, and Needs) are complemented by the two to six most frequently endorsed themes. These represent the strongest similarities among participating programs and suggest practices for program leaders and instructors to inform design of an effective writing-intensive course within an undergraduate public health program.

## 4 Discussion

### 4.1 Summary and trends

Based on data collected from interviews and document reviews of the 30 participating undergraduate public health programs, six primary categories were identified as relevant to support writing instruction and to guide instructors in the improvement of student writing: Approaches, Assignments, Support, Concepts, Context, and Needs. Frequently endorsed themes and codes within these categories contribute to a framework of considerations when designing an effective writing-intensive course within an undergraduate public health program.

Undergraduate public health writing instructors had much to say about their approaches to teaching (as reflected in the Approaches category), the context in which writing-intensive classes are taught (as reflected in the Context category), the assignments that are used (as reflected in the Assignments category), the concepts students struggle with (as reflected in the Concepts category), and the needs to improve instruction (as reflected in the Needs category).

Woven throughout participant interviews and across multiple codes was continued discussion of practices promoting student success. Student success was defined individually by participants to be a combination of academic achievement, career readiness, skill development, confidence, and engagement. In addition to varying definitions, this overarching and integral goal was approached in multiple, diverse ways as participants reported a few common approaches to promote student success.

Another overarching finding from participant interviews is that writing success is associated with how well students receive and employ both discipline-specific instruction and writing-specific instruction. Writing may be a tool to process subject content, and subject content may be used when learning to write. Reading, parsing, and writing scientific articles repeatedly has enormous impacts, along with prolonged practice in managing and using citations. Practicing the integration of technical content into writing—for example, adding statistical evidence to buttress a weak paragraph—is also important. A wide variety of pedagogical approaches can be taken when guiding students to build those three skills. The most endorsed skills include the high-impact educational practices of scaffolding and crafting smaller, but progressive, assignments (33, 34).

Students were also reported to be resistant to the strenuous process of writing, especially after the return to in-person instruction from COVID-related remote learning. Some students had difficulty completing multiple stages of pre-work in order to plan working drafts and then a final product. The suggested reasons for failure were varied. Some instructors relayed that this was a natural decay of skills that come with little-to-no practice between courses, while others claimed students had shifted to short-form communication (e.g., sending SMS and social media direct messages) to such a degree that long essays had become foreign.

Participants also mentioned various partnerships that help students meet course learning objectives, as discussed across the categories of Approaches, Support, and Needs. Instructors stated that discipline-specific writing teacher trainers are needed. Partnerships with university library systems provide students with additional workshops, trainings, and consultations with librarians, writing experts, and teacher trainers. As many of the students’ writing issues are research or technology related, instructors can utilize the expertise of librarians. Instructors mentioned increasing access to librarians and inviting librarians into the classroom to facilitate interactive workshops, including those focused on reference management. Of even greater importance may be policies to build and maintain strong relationships with campus-wide writing centers. Building a writing center within a public health department was proposed as an ideal setting for discipline-specific tutoring and wraparound services. Further, departmental attention to, and support behind, writing-intensive course instructors can increase morale and productivity.

Instructors reported that citation and reference difficulties are widespread. Participants reported students felt citing disrupts their writing flow, which was slow to start, according to instructors. Second, referencing rules are abstruse and may conflict with prior learning. Third, an abundance of style guides, referencing software, and internet citation tools to help guide novice students to acknowledge their sources was noted. However, software tools like reference managers were reported to have steep learning curves, doubling the required learning needed to cite properly. Fourth, many sources being used are relatively modern mediums, such as websites, blogs, and video clips; referencing conventions for these are more complicated than traditional resources (e.g., journal articles and book chapters).

### 4.2 Lessons learned

As the circumstances in which public health is delivered have changed (3, 4, 6), this study suggests, so has the role of preparing students. Programs are going beyond CEPH’s recommendations. Students complete writing assignments, even ones with relatively stringent requirements, like many literature review types, in various ways. Instructors note that students are seeking the flexibility to develop a process that is congruent with previous learning, opportunities to explore topics of their choosing, and the time to experiment with various workflows before committing fully to creating the written product as required.

Assignments should also evolve in response to challenges associated with student writing resilience and to address current trends in the consumption of information. Regardless of the instructional approach, writing assignments should be shorter and more frequent. That is not to say that the long-form essay or literature review does not have its place—these assignments can also be broken down into smaller pieces that facilitate gradual scaffolding and progressive learning, a finding consistent with the integrated approach discussed by Mackenzie (9) and supported by Beard (6). Document summary reviews or annotated bibliographies may be essential to connect these smaller tasks. The challenge is to create assignments that are not overwhelming but still meaningful. Shorter, low-stakes assignments are less intimidating and easier to complete, and therefore, widely utilized. These assignments can also start addressing the academic reading difficulties of students, as noted in findings and consistent with student factors of poor writing in the framework proposed by Lang (15).

Related to writing instruction, reading professional literature and grant applications is an important means of familiarizing students with scientific writing. These findings align with findings, pedagogical theories, and frameworks established in the literature (9, 12, 13, 15, 35–38). Mackenzie mentions, and this study supports, that explicit student support is needed to understand required formatting or required components, especially in teaching grant writing, and reinforces that the lesson is especially important for students with backgrounds historically underrepresented in public health, based on inequities in exposure to such documents (9).

Instructors in this study reported that giving feedback to students is essential. Students need continuous feedback on how they are writing and about what they are writing—both process and content. Many assignments were given, intending to provide specific, continual feedback. As supported in academic literature, the entire process of writing, incorporating feedback, and resubmitting strongly supports students’ writing development, increases performance outcomes, and creates more engagement over the entire process (12, 13, 35–37). Further, Bean and Melzer write that revision is an important component, as it allows the student time to ruminate and reflect on a topic and the time and resources to develop personal writing processes (38). These findings are also consistent with the framework proposed by Lang in highlighting the need for writing instruction to explicitly address student factors, including limited experience with formatting and revision, limited experience reading broadly and related to scientific literature, and insufficient or inadequate critical feedback on writing, and cultural factors, including issues related to diversity in socioeconomic and native language and low literacy value at home, both of which are associated with poor writing among college graduates (15).

### 4.3 Future directions

Future research in three complementary tracks is envisioned. First, subsequent studies can expand upon this work by conducting interviews with faculty and conducting assessments of student products. Using a standard rubric, researchers can unearth any associations between program support and learning outcomes. Also, the interview battery can be standardized to alleviate concerns over reliability. Ideally, an interview would follow the main pedagogical considerations in the framework developed by this work.

A second future research track may expand upon this work by following student progress beyond one course. That is, future studies should also include longitudinal investigation of writing skill development across an undergraduate public health curriculum. Following student outcomes beyond one course is essential to ascertain knowledge and skill retention. Also, this work could provide insights into the effectiveness of the program itself; thoughtful and regular curriculum and program mapping are necessary to develop effective assignments and course sequences that facilitate progressive learning (9, 39). Attention should be paid to the collaborative processes that arise from purposeful development and alignment of assignments, as Mackenzie has noted (9). Encouraging discussion and collaboration between content experts and writing experts is ideal, but more study of how this happens within an undergraduate program is needed.

New challenges are on the horizon. A third future research track would investigate the rise of generative artificial intelligence (AI) and large language models, which offer the prospect of on-demand generated text on any subject (40, 41), in undergraduate public health writing. There are numerous ways in which AI assistance can help students (e.g., brainstorm, outline) and teachers (e.g., plan and streamline), and interest in the effects of AI tools on student outcomes is growing (40, 41). At the same time, AI assistance may detract from students’ learning to write, for example, when students ask AI to fully draft a text from an assignment prompt.

### 4.4 Limitations

There are numerous limitations to the methods employed in this study. Volunteer, social desirability, and researcher biases compromise findings. Definitive conclusions about all public health programs cannot be made, as the sample size is small and represents only 30% of the sampling frame. Almost 75% of the participants worked for colleges in the Northeast or Southeast US. More standardized approaches, such as longer, fully structured interviews and a longer recruitment and sampling time frame, are needed. The addition of quantitative analysis of questionnaire data may also provide rich, complementary data and allow for stronger comparisons among programs, classes, or student groups.

## 5 Conclusion

Within the limitations discussed, including small sample size and substantial geographical sampling biases, this study provides evidence that instructors are adapting well to the undergraduate public health education challenges at hand. Students are increasingly resistant to long-form writing, but instructors are shortening and scaffolding flexible assignments, as well as providing more opportunities for students to personalize writing assignments to promote curiosity and time investment. Based on the framework proposed, undergraduate public health instructors of writing-intensive courses should spend more time having students practice reading, citing, and using scientific literature.

These findings present the first survey of the landscape of writing instruction among public health undergraduate degrees in the US, while meaningfully limited in generalizability to disproportionately represent programs in the Northeast or Southeast US. Findings provide critical considerations to be utilized in design or redesign of writing-intensive courses within public health studies. These findings are supportive context for understanding embedded writing curricula, beyond standardized reporting for accreditation, with implications for individual programs in meeting student needs, while improving the writing skills of future public health workforce.

## Data Availability

The original contributions presented in the study are included in the article/Supplementary material, further inquiries can be directed to the corresponding author.
