# Supplementary material for: The landscape of undergraduate public health writing instruction: a qualitative assessment
Source: Front Public Health. 2026 Apr 1;14:1737041. doi: 10.3389/fpubh.2026.1737041 (PMC13079281; doi:10.3389/fpubh.2026.1737041)
Supplement: Supplementary file 1 [file Table_1.docx]

Appendix A. CEPH-accredited Undergraduate Public Health Degree Offerings

|  | **Program** | **Degree** | **Concentration** |
| --- | --- | --- | --- |
| US | Albany College of Pharmacy and Health Sciences | BS | Public Health |
| US | American University | BA/BS | Public Health |
| LB | American University of Beirut | BA | Health Communication |
| LB | American University of Beirut | BS | Environmental Health |
| US | Andrews University | BSPH | Environmental Health |
| US | Andrews University | BSPH | Social & Behavioral Health Sciences |
| US | Appalachian State University | BS | Public Health |
| US | Arcadia University | BSPH | Global Health |
| US | Baylor University | BSPH | Community Health Education |
| US | Brigham Young University | BS | Health Promotion |
| US | Brigham Young University | BS | Health Science |
| US | Brigham Young University | BS | Environmental & Occupational Health |
| US | Brigham Young University | BS | Epidemiology |
| US | Brown University | AB | Public Health |
| US | California Baptist University - Online | BSPH | Generalist |
| US | California State University, Northridge | BS | Public Health |
| US | California State University, San Bernardino | BS | Public Health Education |
| US | Central Michigan University | BA/BS/ BAA | Public Health Education |
| US | Charles R. Drew University of Medicine and Science | BSPH | Urban Health Disparities |
| US | Clemson University | BS | Pre-Professional Health |
| US | Clemson University | BS | Health Administration |
| US | Clemson University | BS | Health Promotion & Education |
| US | Coastal Carolina University | BS | Public Health |
| US | Coastal Carolina University | BS | Public Health: Community Health |
| US | Coastal Carolina University | BS | Public Health: Dietetics |
| US | Coastal Carolina University | BS | Public Health: Health Administration |
| US | Coastal Carolina University | BS | Public Health: Health Literacy |
| US | Drexel University | BSPH | Public Health |
| US | Drexel University | BA | Global Health |
| US | East Carolina University SBP | BSPH | Pre-Health Professions |
| US | East Carolina University SBP | BSPH | Community Health Education |
| US | East Carolina University SBP | BSPH | Worksite Health Promotion |
| US | East Stroudsburg University | BSPH | Community Health |
| US | East Stroudsburg University | BSPH | Health Service Administration |
| US | East Tennessee State University | BSEH | Environmental Health |
| US | East Tennessee State University | BS | Community Health |
| US | East Tennessee State University | BS | Health Administration |
| US | Eastern Kentucky University | BS | Health Promotion |
| US | Eastern Kentucky University | BS | Pre-professional |
| US | George Mason University | BS | Community Health |
| US | George Washington University | BS | Public Health |
| US | Georgia Southern University | BSPH | Health Education & Promotion |
| US | Georgia Southern University | BSPH | Global Health |
| US | Georgia Southern University | BSPH | Environmental Health Sciences |
| US | Georgia State University | BS | Public Health |
| US | Hawaii Pacific University | BSPH | Generalist |
| US | Illinois State University | BS | Health Promotion & Education-Community Health Promotion |
| US | Indiana University - Purdue University Indianapolis | BSPH | Community Health |
| US | Indiana University - Purdue University Indianapolis | BSPH | Global Health |
| US | Indiana University - Purdue University Indianapolis | BSPH | Epidemiology |
| US | Indiana University at Bloomington | BSPH | Community Health |
| US | Indiana University at Bloomington | BSPH | Epidemiology |
| US | Indiana University at Bloomington | BSPH | Environmental Health |
| US | Indiana University at Bloomington | BSPH | Fitness & Wellness |
| US | Kent State University | BSPH | Global Health |
| US | Kent State University | BSPH | Health Services Administration |
| US | Kent State University | BSPH | Pre-Medicine, Dentistry, Osteopathy |
| US | Kent State University | BSPH | Community Health Outreach & Development |
| US | Kent State University | BSPH | Clinical Trials Research |
| US | Kent State University | BSPH | Allied Health |
| US | La Salle University | BSPH | Health Education |
| US | Liberty University | BS | Health Promotion |
| US | Liberty University | BS | Pre-Clinical |
| US | Louisiana State University Health Sciences Center | BSPH | Public Health |
| US | Loyola University Chicago | BSPH | Generalist |
| US | Mercer University | BSPH | Diverse Populations & Health Equity |
| US | Montclair State University | BS | Community Health Education |
| US | Montclair State University | BS | Health Systems Administration & Policy |
| TW | National Taiwan University | BS | Epidemiology & Preventive Medicine |
| TW | National Taiwan University | BS | Biostatistics |
| TW | National Taiwan University | BS | Health Policy & Management & Health Behaviors & Community Sciences |
| TW | National Taiwan University | BS | Environmental Health Sciences |
| TW | National Taiwan University | BS | Occupational Health |
| TW | National Taiwan University | BS | Global Health |
| US | New Mexico State University | BPH | Health Behavior & Health Promotion |
| US | Northeastern University | BS | Health Science |
| US | Nova Southeastern University | BSPH | Generalist |
| US | Ohio State University | BSPH | Public Health Sociology |
| US | Ohio State University | BSPH | Environmental Public Health |
| US | Oregon Health & Science University/Portland State University | BA/BS | Public Health Studies: Healthy Aging |
| US | Oregon Health & Science University/Portland State University | BA/BS | Public Health Studies: Community Health Promotion |
| US | Oregon Health & Science University/Portland State University | BA/BS | Public Health Studies: Pre-Clinical Health Science |
| US | Oregon Health & Science University/Portland State University | BA/BS | Public Health Studies: School Health Educator |
| US | Oregon Health & Science University/Portland State University | BA/BS | Public Health Studies: Health Administration |
| US | Oregon State University | BS | Health Management & Policy |
| US | Oregon State University | BS | Health Promotion & Health Behavior |
| US | Rutgers, The State University of New Jersey SBP | BS | Public Health |
| US | Saint Louis University | BS | Public Health |
| US | Salisbury University | BS | Public Health |
| US | San Diego State University | BS | Public Health |
| US | San Francisco State University | BS | Community Health Education |
| US | Simmons University | BS | Generalist |
| CA | Simon Fraser University | BA | Health Sciences: General Studies |
| CA | Simon Fraser University | BSc | Health Sciences: Population & Quantitative Health |
| CA | Simon Fraser University | BSc | Health Sciences: Life Sciences |
| US | Southern Connecticut State University | BS | Public Health |
| US | Southern Illinois University, Edwardsville | BS | Public Health |
| US | St. Catherine University | BA/BS | Community Health Worker |
| US | St. Catherine University | BA/BS | Health Science |
| US | St. Catherine University | BA/BS | Public Policy & Social Sciences |
| US | SUNY - Cortland | BS | Community Health |
| US | SUNY - Old Westbury | BS | Health & Society |
| US | Syracuse University | BS | Addiction Prevention |
| US | Syracuse University | BS | Community Health Education |
| US | Syracuse University | BS | Health & Society |
| US | Syracuse University | BS | Healthcare Management |
| US | Temple University | BS | Public Health |
| US | Tennessee State University | BS | Public Health |
| US | Texas A&M Health Science Center | BS | Public Health |
| US | Tulane University | BSPH | Public Health |
| US | University at Albany - SUNY | BS | Public Health |
| US | University at Buffalo - SUNY | BS | General Public Health |
| US | University of Alabama at Birmingham | BS | Environmental Health |
| US | University of Alabama at Birmingham | BS | General Public Health |
| US | University of Alabama at Birmingham | BS | Global Health |
| US | University of Arizona | BS | Public Health |
| US | University of Arizona | BA | Wellness & Health Promotion Practice: Health & Wellness |
| US | University of Arizona | BA | Wellness & Health Promotion Practice: Aging & Population Health |
| US | University of Arizona | BA | Wellness & Health Promotion Practice: Health Education |
| US | University of Arkansas SBP | BSPH | Public Health |
| US | University of California, Berkeley | BA | Public Health |
| US | University of California, Irvine | BA | Public Health Policy |
| US | University of California, Irvine | BS | Public Health Sciences |
| US | University of Florida | BPH | Public Health |
| US | University of Georgia | BS | Environmental Health Science |
| US | University of Georgia | BS | Health Promotion |
| US | University of Hawaiʻi at Mānoa | BA | Public Health |
| US | University of Illinois at Chicago | BA/BS | Public Health |
| US | University of Iowa | BA/BS | Public Health |
| US | University of Kentucky | BPH | Public Health |
| US | University of Louisville | BA/BS | Public Health |
| US | University of Louisville | BA/BS | Public Health: Social Justice & Health Equity |
| US | University of Louisville | BS | Public Health: Professional Health Studies |
| US | University of Maryland | BS | Public Health Science |
| US | University of Maryland | BS | Community Health |
| US | University of Massachusetts Amherst | BS | Public Health |
| US | University of Memphis | BSPH | General Public Health |
| US | University of Miami SBP | BS | Public Health |
| US | University of Michigan | BS | Public Health Sciences |
| US | University of Michigan | BA | Community & Global Public Health |
| US | University of Michigan, Flint | BSPH | Health Education |
| US | University of Missouri - Columbia | BHS | Public Health |
| US | University of Nebraska at Omaha | BSPH | Public Health |
| US | University of Nevada, Las Vegas | BS | Public Health |
| US | University of Nevada, Reno | BS | Community Health Sciences, Public Health |
| US | University of North Carolina, Chapel Hill | BSPH | Biostatistics |
| US | University of North Carolina, Chapel Hill | BSPH | Environmental Sciences & Engineering |
| US | University of North Carolina, Chapel Hill | BSPH | Health Policy & Management |
| US | University of North Carolina, Chapel Hill | BSPH | Nutrition Science & Research |
| US | University of North Carolina, Chapel Hill | BSPH | Nutrition, Health, & Society |
| US | University of North Carolina, Charlotte | BSPH | Community Health Practice |
| US | University of North Carolina, Wilmington | BSPH | Community Health Education |
| US | University of North Carolina, Wilmington | BSPH | Gerontology |
| US | University of North Carolina, Wilmington | BSPH | Pre-Clinical |
| US | University of North Carolina, Wilmington | BSPH | Global Health |
| US | University of North Florida | BSH | Public Health |
| US | University of Oklahoma Health Sciences Center | BPH | Public Health |
| US | University of Pittsburgh | BSPH | Public Health |
| US | University of South Carolina | BA/BS | Public Health |
| US | University of South Florida | BSPH | Public Health |
| US | University of Southern Maine | BSPH | Generalist |
| US | University of Southern Mississippi | BSPH | Health Education |
| US | University of Southern Mississippi | BSPH | Health Policy & Administration |
| US | University of Southern Mississippi | BSPH | Allied Health |
| US | University of Washington | BS | Environmental Health |
| US | University of Washington | BA/BS | Public Health: Global Health |
| US | University of Wisconsin, La Crosse | BS | Community Health Education |
| US | University of Wisconsin, Milwaukee | BSPH | Public Health |
| US | Virginia Tech | BSPH | Public Health |
| US | West Virginia University | BS | Public Health |
| US | West Virginia University | BS | Health Services, Management & Leadership |
| US | Western Connecticut State University | BS | Allied Health Professions |
| US | Western Connecticut State University | BS | Community Health |
| US | Western Connecticut State University | BS | Holistic & Integrative Health |
| US | Western Connecticut State University | BS | Wellness Management |
| US | Western Kentucky University | BSPH | Generalist |
| US | Western Washington University | BS | Behavioral & Community Health |
| US | Western Washington University | BS | Population & Global Health |
| US | William Paterson University | BSPH | Health Promotion |
